# My Family Stands Behind Me: Moderated Mediation Model of Family Support and Work Engagement

**DOI:** 10.3390/ejihpe11020024

**Published:** 2021-03-30

**Authors:** Humaira Erum, Ghulam Abid, Aizza Anwar, Muhammad Fazal Ijaz, Daisy Mui Hung Kee

**Affiliations:** 1 Department of Management Sciences, National University of Modern Languages, Lahore, Punjab 54000, Pakistan; hhamid@numl.edu.pk; 2 School of Business Administration, National College of Business Administration & Economics, Lahore, Punjab 54660, Pakistan; 3 Department of Business Studies, Kinnaird College for Women, Punjab 54000, Pakistan; ghulam.abid@kinnaird.edu.pk; 4 School of Management, Universiti Sains Malaysia Gelugor, Penang 11800, Malaysia; daisy@usm.my; 5 School of Professional Advancement, University of Management and Technology, Lahore, Punjab 54660, Pakistan; 6 Department of Intelligent Mechatronics Engineering, Sejong University, Seoul 05006, Korea; fazal@sejong.ac.kr

**Keywords:** family support, family motivation, work engagement, calling

## Abstract

Family motivation as a mediating mechanism is a novel and under-researched area in the field of positive organizational scholarship. Drawing on Social Exchange Theory (SET), this study empirically validates family motivation as a mediator between family support and work engagement. The process by Hayes (2013) was used to analyze time-lagged data collected from 356 employees of the education sector. Results confirm the mediating role of family motivation in the relationship between family support and work engagement and the moderating role of calling in the relationship between family support and family motivation. This study adds to the literature of family-work enrichment accounts by validating family support as a novel antecedent for family motivation and positive attitudes. The implications of the study are discussed.

## 1. Introduction

“I take comfort in knowing no matter which path I choose, my family stands behind me.”—Benjamin Bratt.

Motivation is an energized stimulation that directs and sustains employee performance and positive behavioral outcomes [1]. It manifests a behavior directed at a particular goal [2]. Motivation is a multifaceted and complex subject in research as different people are motivated for different reasons. Motivation literature into two basic categories; Ref. [1] extrinsic motivation resulting from external rewards or benefits, and [2] intrinsic motivation originating from inherent joy and interest in the activity [3]. The concept of prosocial motivation: the aspiration of benefitting others like colleagues, clients, customers, and/or any specific group of beneficiaries [4]. But the same is called family motivation when the beneficiary of an employee’s work is his/her own family [5]. The family included people who are related biologically, socially, and/or through marriage and adoption [6]. Hence, an employee’s family does not only comprise of spouse and kids, but also grandparents, parents, aunts and uncles, cousins [7]. Family is considered as a perceptual unit based on employees’ distinctive situations and involves everyone who is considered as kin by the employee [7]. This perspective is probably more applicable in eastern regions where collective culture generally prevails and an employee has many dependent members.

Employees usually do not work for themselves but also to provide for their families [5,8]. Therefore, it may be a strong motivational force that keeps them engaged with the organization. This becomes especially relevant when employees have limited job opportunities like in developing regions [9] and face many job stressors [10] which devoid them of pursuing their call, -the inner voice to adopt a profession [11], and intrinsic motivation.

Existing literature provides empirical evidence for family motivation as a significant predictor of energy and job performance [5], productivity [12] and organizational citizenship behavior [OCB], self-efficacy, and affective commitment [13]. The negative effects or dark side of family motivation is also empirically validated in the literature like increasing stress [5], decreasing creativity [12], causing emotional labor and burnout [14], and limiting voice behavior [15]. Moreover, family motivation plays a contingent role in the correlation between work behaviors (like turnover intentions, job performance) and abusive supervision [16]. As regards to antecedents, family financial pressure is supported by the literature as a driver of family motivation [12].

Nevertheless, family as a motivational force to work has remained under focused [17,18]. Family motivation is an evolving construct. Academic and empirical research is required for validating the predictors and consequences of family motivation. It is reported in study that impact of family motivation on important individual outcomes continues to accumulate, thus there is a need to include the antecedents of family motivation” [12]. This theoretical expansion is necessary to develop a key part of the nomological network surrounding family motivation and can help managers and organizations understand when family motivation may be a dominant motive driving employee attitude and behavior [19]. Similarly, it is argued that encourage future researchers to examine factors beyond job performance, such as organizational commitment and organizational citizenship behaviors [5]. Therefore, the current study intends to investigate the determinants of family motivation and the positive attitude resulting from family motivation. Positive attitudes like organizational commitment, work engagement, and job satisfaction have resonated the most among academic researchers over the last two decades [20,21,22,23,24]. Though current literature has validated the antecedents of positive behavior, yet mechanism connecting these antecedents to behaviors is required to be established [25,26]. Moreover, there is a need to investigate how and why these antecedents lead to these positive behaviors [27,28]. 

This study also responds to these calls. This study specifically focuses on (1) if family support is the antecedent of family motivation. (2) Whether family motivation results in work engagement? (3) Examining the mediating mechanism of family motivation between family support and work engagement and (4) testing moderating role of calling between family support and family motivation and work engagement.

## 2. Literature Review and Hypotheses

### 2.1. Family Support and Family Motivation

Social support means the “exchange of resources between at least two individuals perceived by the provider or the recipient to be intended to enhance the well-being of the recipient” [29] or as “the availability of helping relationships and quality of those relationships” [30] (p. 5). The scholars of social support [31] postulate supportive relations play an important role in an individual life to enhance his/her well-being and increase his/her ability to deal with setbacks in life. The individual gains resources like assistance, advice, and compassion through supportive relations [32]. Work-life researchers have emphasized that social support practiced as family and co-workers support results in lesser work-family conflicts and greater work-family enrichment [17,33].

Family support which stems from social support refers to concern and commitment among family members and how they support each other [34]. Family offers both emotional and instrumental support. Emotional family support means how family members make each other feel cared whereas instrumental family support signifies physical/tangible ways to help family members in daily tasks [35]. The relevance of family support resulting in positive work outcomes is established by many work-family enrichments [36,37] work-family conflict [33,38,39] and stress management studies [40]. Family support buffers against work-related stress and burnout as it provides emotional resources like keeping an employee’s morale up by encouraging and guiding him and listening to his/her experiences at work. The family also offers instrumental support like helping with household chores and sharing child care so that employee can focus more on work-related tasks, can have positive experiences and perform better in multiple roles [36,41]. Family support helps employees achieve life goals and aspirations [37] and has a positive association with employee motivation [42]. On the basis of this evidence, the current research postulates family support as the antecedent of family motivation.

When the employee has close bonding with his/her spouse/children/relatives, these individuals help the employee emotionally by counseling, guiding, and motivating him/her. Moreover, the family provides physical help in sharing household, children, and social responsibilities. So, the employee is more concerned, attached, and inclined towards his/her family and exerts energy and effort to support the members of his/her family. On the contrary, if the employee does not get the guidance, advice from family members, does not share problems or worries, and/or does not get support physically in daily tasks, that is, he/she is not connected to family, it is less likely that he makes any effort to do a job for supporting family members leading us to hypothesize [43].

**Hypothesis** **1** **(H1).** *Family support is positively related to family motivation*.

### 2.2. Family Motivation and Work Engagement

Kahn’s seminal research has laid foundations for an exploration of “work engagement as a psychological state” which was referred to initially as “the harnessing of organization members’ selves to their work roles” [44] (p. 694). Most of the researchers consider that work engagement is the psychological presence that indicates the degree of employee’s attentiveness, engrossment, and focus to his/her work role activities [19,45]. These authors suggest work engagement as a multidimensional construct comprising of physical and cognitive mechanisms. Work engagement based on three critical elements: attention, absorption, and energy [44,45]. Attention refers to material resources within a person that can be applied to a given task. Absorption is defined as the capacity and capability to apply these resources intensely whereas energy means the physical effort that employees put forth towards task accomplishment [44].

Recent empirical study found that the perception of organizational support, thriving at work, employee calling and flourishing positively enhances work engagement [46,47]. Another study found that colleague support, supervisor support, and possibilities for professional development boost work engagement of employees [48]. Furthermore, it is identified that employees are most probably engaged with the work when they are thriving, prosocially motivated in civil environment [49].

Almost all the definitions in work engagement literature incorporate the notion of energy. An employee fulfills his need of supporting and benefitting family members by doing a job so this need fulfillment pushes him to exert effort. Energy has emotions as its source and relational interactions are the basis of emotional energy [50]. This concept provides a conceptual basis to link family motivation with work engagement. Family motivation makes the point that an employee works to benefit the family due to emotional bonding [51] and kinship [7]. Family motivation energizes employee’s efforts to result in positive outcomes [5]. Therefore, we argue that;

**Hypothesis** **2** **(H2).** *Family motivation is positively associated with work engagement*.

An employee’s family sharing a sense of belongingness, relatedness, compassion and care pushes an employee to reciprocate positively by doing something beneficial for the family. Employees’ job provides an opportunity for him/her to support the family and provide for their financial and social needs. Also, instrumental and emotional support provided by family-members buffer against the work stress, helps in stress management by counseling and guiding the employee in low times. So when the family motivation is high, employees value the work more as it is associated with their personal value of supporting the family, which inculcates persistence [43] to work harder and longer. This leads to higher levels of work engagement.

**Hypothesis** **3** **(H3).** *Family motivation mediates the relationship between family support and work engagement*.

### 2.3. Calling as Moderator

Calling is defined as an individual’s work with a sense of resolution and purpose [52]. Calling refer as the urge that becomes a reason for an individual’s existence [53,54]. Career calling means “a transcendent summons, experienced as originating beyond the self, to approach a particular life role in a manner oriented toward demonstrating or deriving a sense of purpose or meaningfulness that holds other-oriented values and goals as primary sources of motivation.” [55] (p. 427). Calling has three dimensions: “identification and person-environment fit”; “sense and meaning and value-driven behavior”, and “transcendent guiding force” [11]. Therefore, there is a consensus among modern scholars that calling is action-oriented and prosocially focused. It advocates a meaning or mission [56]. Individuals with calling orientation of work identify themselves with their work and express their passion and excitement through their work [54]. Called people to experience both selves–congruence and outer congruence. Self-congruence means coherence between the ideal and the real self, that is, “I am what I want to be”, whereas outer congruence refers to compatibility between an individual’s interests, required job skills, and job-related tasks [11]. 

Moreover, researchers of calling agree that there is a difference between recognizing a call, and experiencing a call [54]. Recognizing a calling means the extent to which one’s work expresses his/her calling. Experiencing a calling means the extent to which a particular job endorses one’s felt calling. More recently, researchers [57,58] propose that just the identification or recognition of calling does not produce positive outcomes at the workplace. Identification/recognition of calling relates to following a particular work/career by an employee just because it matches his/her prosocially-oriented purpose of life. But this is not true for everyone. Many people pursue careers that do not match their personal ambitions and preferences because of personal and contextual hurdles [59]. If a person does not live his/her call, his/her over-all life satisfaction and well-being may decline as a result [60]. Some people do not experience calling in their work due to many factors like unfavorable work environment, economic conditions, punitive supervision, poverty, discriminatory practices, workplace mobbing, and unemployment [55,61]. These constraints limit a person’s ability to search for a job that matches his/her calling [62]. In such scenarios, when an individual remains unable to live his/her call, he feels dissatisfied and stressed, family support plays its role by counseling, encouraging, guiding the individual to motivate him/her to work for his family. ‘Working for family’ gives meaning and purpose and serve as work orientation. Therefore, we argue that family support compensates for lack of calling and motivates the employees to work by giving his work the purpose of supporting a family. Hence, we can say that;

**Hypothesis** **4** **(H4).** *Employee calling moderates the relationship between family support and family motivation such that when employee calling is low, the relationship between family support and family motivation is strong*.

**Hypothesis** **4a** **(H4a).** *Employee calling moderates the mediated relationship between family support and work engagement through family motivation such that when employee calling is low, this mediated relationship is strong*.

**Figure 1:**
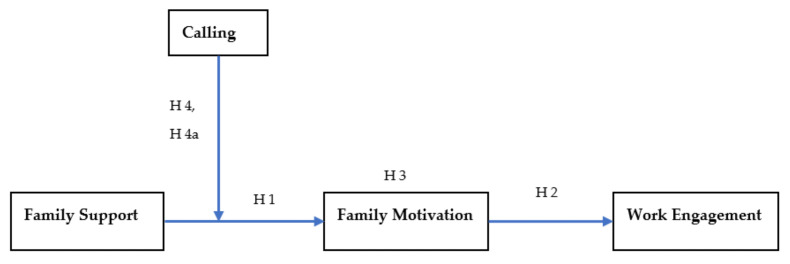
Moderated Mediation Model.

The relationships among variables have graphically representative in the theoretical framework in Figure 1.

## 3. Methods

### 3.1. Participants and Procedure

The data was collected from the education sector, mainly from public and private sector employees serving in schools and colleges, located in Lahore, Pakistan during first quarter of 2020. The factors like high job demands, low compensation, unattractive career, and forced adoption due to unavailability of better opportunities make teaching a profession where employees were expected to experience low extrinsic and intrinsic motivation, low calling and high family motivation. That is why it was considered appropriate to investigate and empirically test the phenomenon of family motivation in this population. Convenience sampling was used to draw the sample, keeping in view the study’s exploratory nature and non-availability of exact size of the population. Informed consent was obtained from all participants involved in the study and the objective of the study was shared with all the participants.

The recommended sample size for behavioral research is between 30 and 500 [63] (pp. 307–308) [64] (p. 163). As a rule of thumb, the sample, 10 to 25 times more than the number of variables in the study is considered appropriate in multivariate analysis [64]. This study has 5 variables. So, based on the rule of thumb [64], the minimum sample consisting of 125 (5 × 25) is adequate. Based on the above recommendations by scholars, we targeted a sample of 500 employees from the education sector. The complete sample profile is given in Table 1.

A team of 10 research students was recruited. These students were trained for the intricacies of the research and data collection process so that level of researcher interference can be kept to a minimum. They collected data by visiting different schools, colleges and institutes. Before handing over the questionnaire, the students’ elucidated the study objective, ensured the anonymity of data, and acquired consent to participate from the respondents. The confidentiality of responses was guaranteed through a letter from the institution. Provided with the reliance on the validated questionnaire, those who agreed to join in the study were sampled in two time-lagged meetings (7 days lapsed) to minimize common method bias as recommended [65]. From the organization’s paid time, a 20 min meeting was arranged with employees for each session. Participants completed the survey in two waves. At Time 1 (T1), a self-reported response was provided by employees about family support, calling, and demographics. At Time 2 (T2), they answered questions about family motivation, intrinsic motivation, and work engagement seven days later. At T1, we contacted 500 employees. Participating in the first survey were 432 employees, with a 86% response rate. Completing the second survey were 390 employees. Missing data on the study variables reduced the sample size for the main analysis to 354 employees, generating a response rate of 70.8%. The respondents were instructed to write self-identifiable code or name to cross match the T1 and T2 response.

### 3.2. Sample Profile

The sample profile presented in Table 1 indicates that the majority (70%) were females that participated in this survey. 64% employees held Master’s degree followed by 29% M. Phil. degree holders. The majority of respondents (61%) were married. The average age of respondents is 35 years, calculated as mid-point of each category.

### 3.3. Measurement of Variables

Family support as a dimension of social support was measured using 4-items, 5-point Likert scale [66]. The scale ranged from “1 = strongly disagree to 5 = strongly agree” including sample items like “My family really tries to help me” and “I get emotional help and support I need from my family.” A 5-item scale was used to collect data of family motivation on 5-point Likert scale [67]. The scale ranged from “1 = strongly disagree to 5 = strongly agree” including sample items like “My family benefits from my job?” and “It is important for me to do good for my family?” Work engagement represents employee’s psychological presence in work role and captures the degree to which employees are attentive, engrossed and focused to their work role activities [45]. It was measured using a 9-item scale [68,69] including items like “At my job, I feel strong and vigorous.” and “I am immersed in my work.” on five-point Likert scale ranging between “1 = never to 5 = very often.” Calling means “work that a person perceives as [her or] his purpose in life” [52] (p. 160). A 9-item scale [70] was used to measure calling including items like “I identify with my work.” and “An inner feeling is guiding me in doing my job.” on 5-point Likert scale ranging between “1 = strongly disagree to 5 = strongly agree.”

## 4. Results

### 4.1. Preliminary Analysis

Primary assistance for proposed hypotheses is sought using bivariate correlation among the variables. Table 2 presents the descriptive statistics and correlation among variables which provides support for further hypothesis testing.

Positive and significant correlation was found among the study variables. Family support is positively and significantly related to family motivation (r = 0.23, *p* < 0.01), calling (r = 0.34, *p* < 0.01), and work engagement (r = 0.31, *p* < 0.01). Correlation analysis also shows a positive and significant relationship between family motivation and calling (r = 0.21, *p* < 0.01), family motivation and work engagement (r = 0.31, *p* < 0.01), and calling and work engagement (r = 0.53, *p* < 0.01) which provides support for direct relationships in the model. 

### 4.2. Validity Assessment

Before testing the association among study variables, construct validity was tested. It assured whether the instrument taps the concepts as theorized. Construct validity is tested through convergent and discriminant validity [71]. Convergent and discriminant validity of the measures were tested, as well as the inter-correlation among variables. SPSS version 20 was used for the analysis. Regarding convergent validity it is suggested the correlations between the factors of a particular construct should be higher [greater than 0.5 is acceptable] [72]. To assess the discriminant validity, correlations between factors of the distinct construct should be low [should be less than 0.5]. The results show that the correlation between factors of different constructs ranged from 0.002 to 0.459 whereas the correlation between factors of the same constructs ranged from 0.496 to 0.701.

To assess the construct validity, convergent and discriminant validity were estimated. A construct factor loadings, composite reliability (CR) and the average variance extracted (AVE) are required to be calculated [71]. The acceptance criteria are that factor loadings for each variable should be greater than 0.60, composite reliability should be greater than 0.70 and average variance extracted should be greater than 0.50. For discriminant validity, ‘square root of AVE of each construct should be greater than the correlations of this construct to all the other constructs [72]. The results, presented in Table 3, show that the factor loadings are greater than 0.64. Moreover, all variables have CR and AVE greater than 0.70 and 0.50, respectively. Thus, fulfilling the criteria for convergent validity.

Confirmatory Factor Analyses (CFA) was performed using AMOS version 21. Results revealed that the hypothesized four factor model (CMIN/Df = 2.4; RMSEA = 0.06; CFI = 0.93; TLI = 0.92, GFI = 0.90) substantially fits the data better than the other models like, for example, two-factor model A (CMIN/Df = 4.9; RMSEA = 0.10; CFI = 0.81; TLI = 0.79, GFI = 0.82) and single factor model C (CMIN/Df = 11.1; RMSEA = 0.16; CFI = 0.51; TLI = 0.45, GFI = 0.61) (see Table 4).

### 4.3. Hypotheses Testing

To test the moderated-mediated model (Figure 1), PROCESS by Hayes macro in SPSS, Model 4, was used with a 95% bias-corrected confidence interval on a 5000 bootstrap sample. Table 5 displays the results under the title “without moderation”. As proposed, family support is positively related to family motivation with β = 0.16, *p* < 0.001, CI 95% [0.10, 0.22]. The range of upper and lower bound excludes zero, consequently; H1 is supported. H2 hypothesized that family motivation is positively associated with work engagement with β = 0.21, *p* < 0.001, CI 95% [0.14, 0.28]. Hence, H2 is also supported. H3 proposed that family motivation mediated the relationship between family support and work engagement. The results support H 3 as indirect effect is significant with β = 0.03, CI 95% [0.01, 0.06].

Results indicated in Table 5, titled as “with moderation” suggest that family support and family motivation have significant positive relationship with β = 0.56, *p* < 0.05, CI 95% [0.27, 0.85]. Similarly, calling and family motivation is positively and significantly related as depicted by β = 0.73, *p* < 0.05, CI 95% [0.37, 1.10]. As predicted in H4, the interaction effect of family support and calling has significant negative relationship as indicated by β = −0.11, *p* < 0.05, CI 95% [−0.17, −0.04]. Therefore, H4 is supported.

The moderated mediated analysis was performed using Model 7 of PROCESS by Hayes macro. The results are presented in Table 5. There is a positive association between family support and work engagement (β = 0.15, *p* < 0.01). Similarly, the positive relationship between family motivation and work engagement is also significant (β = 0.21, *p* < 0.01).

In Table 5, the significant indirect effect of family support on work engagement was found at a low level of calling with β = 0.04, CI 95% [0.02, 0.06] and at a moderate level of calling with β = 0.02, CI 95% [0.01, 0.04] as confidence interval does not span zero. But at a higher level of calling, the mediated relationship of family support and work engagement is insignificant with β = 0.01, CI 95% [−0.01, 0.03] which supports H4. The moderated mediation index indicates that the indirect effect of family motivation on work engagement regressed on family support x calling was significant as 95% CI has not included zero (β = −0.02 [LLCI = −0.04 ULCI = −0.01]). So, supporting H4a.

## 5. Discussion and Implication

The theoretical model based on social exchange theory [SET], tested family support as antecedent and work engagement as consequences of family motivation. Calling was tested as a moderator between family support and family motivation.

Results show that all of the study hypotheses were supported and are in line with existing literature without any significant contradiction. Existing studies provide evidence that family support helps employees achieve life goals and aspirations [18] and is positively associated with employee motivation [42] which is in line with our results that family support is positively related to family motivation. On the other hand, we also found that family motivation results in enhancing positive attitude i.e., work engagement which is also in line with existing literature [46]. The relationship between family motivation and work engagement is novel tested in this study, though we get the evidence from the literature that family motivation plays a significant role in boosting employee energy [5]. Since energy is also one of the dimensions of work engagement [68] so as expected, our hypothesis was supported. Results also show that calling moderates the relationship between family support and family motivation in such a way that when calling is low, the relationship between family support and family motivation is stronger. This is a unique finding of the current study, which is not empirically tested and supported in the existing literature.

The current study gains strength by adopting methodological measures to reduce common method bias [65]. First, anonymity was assured by assigning a self-identifiable code to the respondents. Secondly, the data for different variables were collected at three time periods separated by 14 days.

Our research has provided useful insight related to the phenomenon of family motivation. From a theoretical perspective, it has contributed to Positive Organization Behavior [POB], positive psychology and adds to family-work literature. Our research responded to the call of paper [72] to extend the application of POB in general and positive constructs in particular by expanding the boundaries of these constructs to other contexts. They argue that positivity is culturally sensitive so U.S.-based positive models are required to be tested in other cultures for a better understanding of the positive constructs. The present study fulfills this demand by researching family motivation in South Asian culture.

Drifting from existing literature, we used the reciprocation lens of social exchange theory to explain the mechanism of family motivation and thus providing empirical evidence for the theory from family enrichment literature. Existing literature on family motivation is very limited. This research added to the existing body of literature by providing empirical evidence for several novel relationships regarding antecedents and consequences of family motivation and its mechanism. For example, family support is recognized as the new antecedent of family motivation. Similarly, work engagement is validated as the novel consequence of family motivation.

Practically, this research is useful for managers, human resource trainers, and consultants. First of all, we found that family motivation is a key driver to work for employees’ positive attitude. Sociologists and psychologists should highlight the importance of family support as it does not only provide a conducive environment for developing family motivation but also buffers against stress and helps in stress management which ultimately results in positive outcomes at the workplace. Family support also compensates for an employee calling and enhances his/her motivation. Secondly, it is also very important that human resource trainers/managers must consider employee’s orientation to motivation and not just the level of motivation. Third, family motivation is advantageous because it is conducive to cognitive job crafting and does not require extensive resources and effort so, leaders by reminding the employees of how their jobs are contributing to the family can reframe their work orientation as more meaningful and motivating. Fourth, family motivation is more consistent across different contexts/jobs, so companies offering more complex and tough jobs may benefit by alluring to employees’ family motivation like family picnics, ‘bring your child to work’ days allow employees to integrate their family life in their work. Fifth, managers must understand the nature of their employee’s family motivation and provide them the opportunities to meet family needs. For example, females and/or employees with high family motivation may show more engagement and reduced turnover intention if they are allowed flextime, working in shifts and/or daycare facility at the workplace so that they can better manage work and family life.

### Limitations and Future Directions

Following limitations must be considered before using the results of the current study and future research may address these limitations. First, this study used a non-probability sampling technique (purposive sampling) so care must be taken in the generalizability of results. We suggest future studies should use probability sampling design in different cultures, contexts, and settings to include participants from different sectors. Second, the use of cross-sectional and correlational design does not allow to draw causal inferences among study variables. Future research must take up longitudinal and experimental studies to examine causation among study variables. Third, the study respondents were included from schools and colleges located in one city so the county-wide context is not represented by the sample. Therefore, future research must replicate this study in other regions to enhance the generalizability of our findings. Fourth, because of the exploratory nature of the study, we did not use controls (like age, gender, marital status, etc.) in the overall model for data analysis. Future researchers may analyze the model, including controls. Fifth, the sample of the study consisted of 70% females, which may raise concerns for generalizability of study for both genders.

## 6. Conclusions

Based on this research results, family support is empirically validated as a novel antecedent of family motivation and also compensates employee calls. Similarly, family motivation is recognized as the mediating mechanism between family support and work engagement. This research is at a nascent stage and needs further empirical and theoretical investigation.

## Figures and Tables

**Table 1:** Participants’ Profile.

	Frequency	Percentage (%)
**Gender**		
Female	250	70.2%
Male	106	29.8%
**Qualification**		
Graduate	19	5.3%
Masters	228	64.0%
MPhil/MS	104	29.2%
Doctorate	5	1.4%
**Marital Status**		
Unmarried	120	33.7%
Married	218	61.2%
Divorced	6	1.7%
Widowed	5	1.4%
Separated	7	2.0%
**Age**		
Below 30	137	38.5%
31–35	77	21.6%
36–40	73	20.5%
41–45	30	8.4%
46–50	18	5.1%
Above 50	21	5.9%
**Work Experience**		
Less than 5 years	115	32.3%
6 to 10 yrs	103	28.9%
11 to 15 yrs	69	19.4%
16 to 20 yrs	40	11.2%
More than 20 years	29	8.1%

**Table 2:** Descriptive and Correlation.

	Mean	St. Dev.	FS	FM	Call
FS	5.87	1.09	1		
FM	4.09	0.79	0.23 **	1	
Call	4.31	0.59	0.34 **	0.21 **	1
WE	4.27	0.65	0.31 **	0.31 **	0.53 **

Note: ** *p* < 0.01; FS = Family Support, FM = Family Motivation, Call = Calling, WE = Work Engagement.

**Table 3:** Convergent and Discriminant Validity.

	CR	AVE	MSV	MaxR(H)	FM	FS	Call	WE
**FM**	0.869	0.574	0.133	0.891	**0.758**			
**FS**	0.847	0.652	0.145	0.939	0.283	**0.807**		
**Call**	0.834	0.500	0.381	0.954	0.251	0.381	**0.707**	
**WE**	0.872	0.533	0.381	0.965	0.365	0.343	0.617	**0.730**

Note: Bold values in diagonal represent the squared root estimate of AVE.

**Table 4:** Confirmatory Factor Analysis. Model A; WE & FS Combined. Model B; FM & FS Combined & WE & Calling. Model C; all variables combine as one factor.

Model	CMIN/DF	CFI	GFI	TLI	IFI	RMSEA
Full Measurement Model	2.4	0.93	0.90	0.92	0.93	0.06
Model A (3 factor)	19.7	1.0	1.0		1.0	0.23
Model B (2 factor)	4.9	0.81	0.82	0.79	0.81	0.10
Model C (1 factor)	11.1	0.51	0.61	0.45	0.52	0.16

**Table 5:** Results of Moderated Mediated Model. Confidence interval range without zero shows a significant relationship.

	Unstandardized Coefficients with Confidence Intervals			
Analysis	Family Motivation (M)		Work Engagement (Y)	
	Coeff.	95% CI	Coeff.	95% CI
**Without Moderation**				
Family Support (X)	0.16	0.10, 0.22	0.15	0.10, 0.20
Family Motivation (M)			0.21	0.14, 0.28
Indirect Effect (X => M => Y)			0.03	0.02, 0.06
R^2^	0.05		0.16	
F	19.29		33.10	
**With Moderation**				
Family Support (X)	0.56	0.27, 0.85	0.15	0.10, 0.20
Family Motivation (M)			0.21	0.14, 0.27
Calling (W)	0.73	0.37, 1.10		
X x W	−0.10	−0.17, −0.04		
R^2^	0.09		0.16	
F	11.31		0.36	
**Conditional Indirect Effects of X on Y**				
	**Values of Calling**	**Effect**	**95% Confidence interval**	
Family Motivation	3.72	0.04	0.02, 0.06	
Family Motivation	4.31	0.02	0.01, 0.04	
Family Motivation	4.91	0.01	−0.01, 0.03	
**Moderated Mediation Index**				
	Index	SE	95% confidence interval	
Family Motivation	−0.02	0.01	−0.04, −0.01	

## Data Availability

The data presented in this study is available on reasonable request from the first author.
